# Multifunctional Nanocomposite Hydrogel with Enhanced Chemodynamic Therapy and Starvation Therapy for Inhibiting Postoperative Tumor Recurrence

**DOI:** 10.3390/ijms252111465

**Published:** 2024-10-25

**Authors:** Zeliang Li, Xiaoxuan Ma

**Affiliations:** 1 Engineering Research Center of Western Resource Innovation Medicine Green Manufacturing, Ministry of Education, School of Chemical Engineering, Northwest University, Xi’an 710069, China; anchenyang@stu.nwu.edu.cn; 2 Shaanxi Key Laboratory of Degradable Biomedical Materials, School of Chemical Engineering, Northwest University, Xi’an 710069, China; 3 Shaanxi R&D Center of Biomaterials and Fermentation Engineering, School of Chemical Engineering, Northwest University, Xi’an 710069, China; 4 Biotechnology & Biomed, Research Institute, Northwest University, Xi’an 710069, China

**Keywords:** hunger therapy, photothermal therapy, postoperative melanoma

## Abstract

Surgical resection is the primary treatment for melanoma; however, preventing tumor recurrence after resection remains a significant clinical challenge. To address this, we developed a multifunctional nanocomposite hydrogel (H-CPG) composed of glucose oxidase (GOx)-coated CuS@PDA@GOx (CPG) nanoparticles, aminated hyaluronic acid (HA-ADH), and oxidized rhizomatous polysaccharides (OBSP), which are interconnected through hydrogen bonds and dynamic Schiff base linkages. In the acidic tumor micro-environment, the hydrogel releases GOx, catalyzing the production of hydrogen peroxide (H_2_O_2_), which enhances chemokinetic activity through a Cu^2+^-mediated Fenton-like reaction. This process generates hydroxyl radicals that intensify oxidative stress and promote macrophage polarization from the M2 to M1 phenotype. This polarization triggers the release of pro-inflammatory cytokines, thereby inhibiting tumor recurrence. Additionally, the hydrogel induces photothermal effects that help eradicate residual bacteria at the wound site. Overall, the H-CPG hydrogel offers a dual mechanism to prevent melanoma recurrence and reduce resistance to monotherapy, presenting a promising strategy for postoperative tumor management.

## 1. Introduction

Surgical resection is one of the most important means applied in clinical medicine [1]. However, melanoma’s aggressive and invasive nature makes recurrence at the wound site common [2,3]. Conventional oncological treatments, such as chemotherapy and radiotherapy, are used to prevent tumor recurrence, but they present challenges. Systemic intravenous chemotherapy struggles to reach and penetrate distant tumor cells and often has a short half-life [4]. Moreover, both chemotherapy and radiotherapy have numerous side effects [5]. Thus, inhibiting tumor recurrence post-surgery and reducing resistance to monotherapy remains a significant challenge.

With advancements in multifunctional nano biomaterials, nanotechnology is becoming a focal point in cancer therapy. Studies have shown that nanomedicines containing copper sulfide (CuS) can be effective for melanoma treatment [6,7]. However, the limitations and potential drug resistance associated with nanomedicines necessitate combining nanotechnology with other therapies. Photothermal therapy (PTT) using a near-infrared laser (NIR) is emerging as a novel treatment option [8,9]. PTT employs a near-infrared (NIR) laser and photo-absorbing agents to generate heat from light energy to “burn” cancer cells [10]. Polydopamine (PDA), known for its biocompatibility and strong photothermal conversion ability [11,12,13], is ideal for PTT materials [14]. Nonetheless, the inflammatory environment induced by PTT can favor melanoma recurrence, and the anti-tumor immune response remains insufficient to prevent recurrence [15,16,17].

Glucose oxidase (GOx) can react with glucose in the tumor microenvironment to produce gluconic acid, thereby lowering pH [18]. Concurrently, hydrogen peroxide produced by GOx decomposition enhances chemodynamic therapy [19,20]. The reduced pH environment also improves H_2_S release [21], inhibiting hydrogen peroxide scavenging enzyme activity and enhancing the hydrogen peroxide cascade reaction. We hypothesize that a multifunctional material combining mild photothermal therapy with starvation therapy could effectively prevent tumor recurrence post-surgery. Hydrogels have long been studied for biomedical drug delivery applications [1], using biomaterials such as chitosan, alginate, hyaluronic acid (HA), and collagen [22]. Rhizome polysaccharide (BSP), extracted from bletilla striata, a traditional Chinese medicine with a history of over 1500 years, has anti-inflammatory and hemostatic properties [23]. BSP’s physical properties, such as good swelling capacity to absorb excess exudate around wounds, make it suitable for hydrogel synthesis.

**Figure 1:**
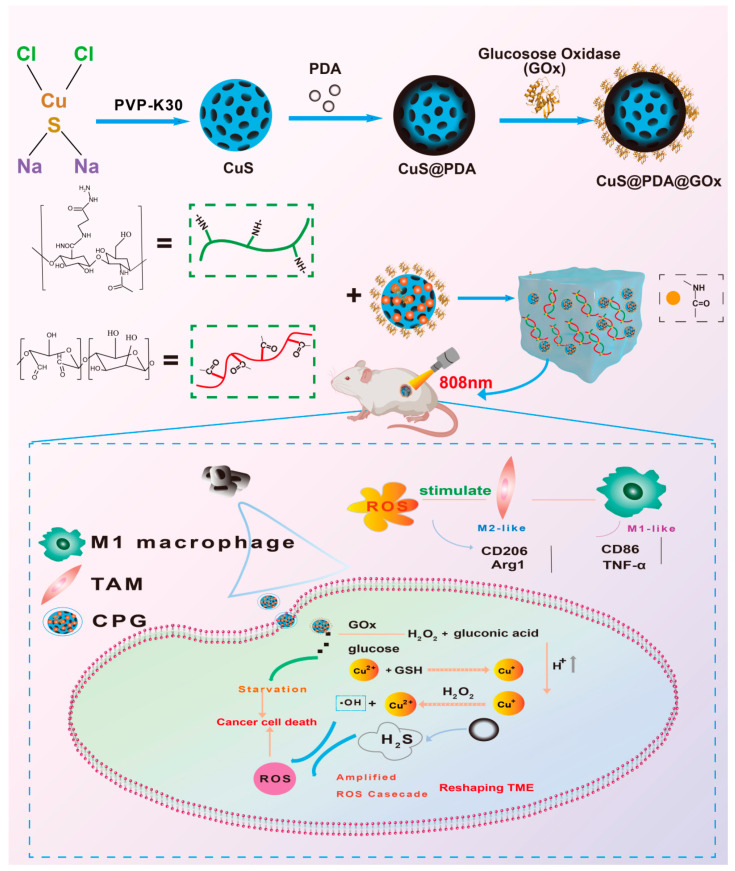
Mechanism diagram of the synthesis of CuS@PDA@GOx nanoparticles and H-CPG hydrogel. Mechanism diagram of recurrence inhibition in postoperative melanoma application of H-CPG hydrogel.

In this study, we designed a multifunctional composite hydrogel (H-CPG) (Figure 1). We prepared CuS via a hydrothermal method, coated it with PDA, and finally encapsulated GOx (CuS@PDA@GOx). The CuS@PDA@GOx (CPG) nanoparticles were cross-linked with HA-ADH and OBSP by means of dynamic Schiff base bonds and hydrogen bonds to form a multifunctional nanocomposite hydrogel (H-CPG). The H-CPG hydrogel effectively reduces bacterial growth on the wound surface while slowly releasing the drug for multiple tumor treatments at predetermined intervals.

The multifunctional hydrogel has the following characteristics. CPG-mediated starvation therapy contributes to glucose catabolism, generating gluconic acid and hydrogen peroxide. On one hand, the lowered pH environment accelerates the release of H_2_S and inhibits hydrogen peroxide scavenging enzyme activity, thus enhancing the chemodynamic treatment of tumor cells. On the other hand, the Fenton-like response of combined Cu^2+^ enhances the oxidative stress level, modulates the immune microenvironment, and stimulates macrophage polarization to a pro-inflammatory phenotype. A multimodal combination therapy is achieved. In conclusion, combination therapy with multifunctional hydrogels offers a promising prospect for inhibiting tumor recurrence after surgery.

## 2. Results and Discussion

### 2.1. Preparation and Characterization of CPG NPs

**Figure 2:**
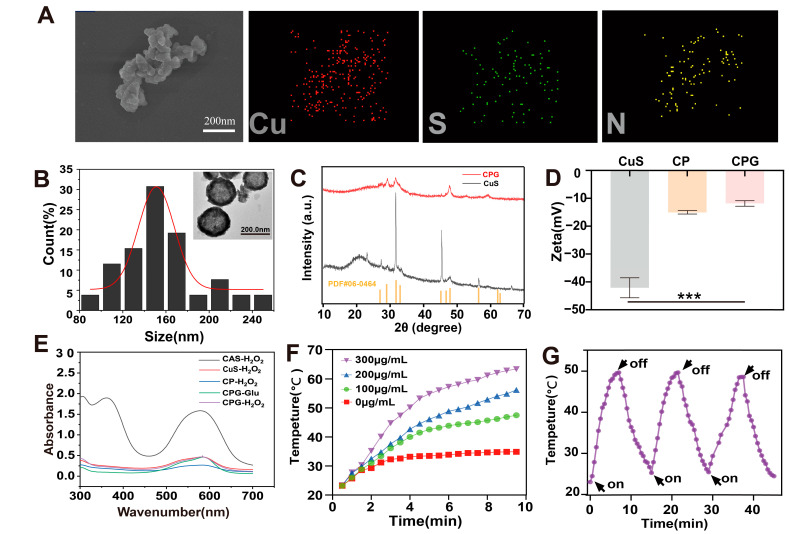
Preparation and characterization of CPG: (**A**) EDS image of CPG. (**B**) Size distribution and TEM image of CPG. (**C**) XRD image of CPG. (**D**) zeta potential maps of CuS, CP, CPG. (**E**) CAS-H_2_O_2_, CuS-H_2_O_2_, CP-H_2_O_2_, CPG-H_2_O_2_, CPG-Glucose (Glu) for the determination of -OH generation. (**F**) Photothermal heating curves for 10 min at different concentrations of CPG. (**G**) Photothermal stability of CPG with 3 on/off cycles. (Mean ± SD (*n* = 3)). *** *p* < 0.001.

We prepared CPG by the hydrothermal method using CuS as the template. Characterization of the CPG morphology was performed by SEM and transmission electron microscopy (TEM). SEM and TEM revealed nanostructures with a size of about 150 nm (Figure 2A,B). Energy-dispersive X-ray spectroscopy (EDS) shows the uniform distribution of the elements Cu, S, and N in the CPG structure (Figure 2A), proving that our coatings are successful. X-ray diffraction (XRD) patterns of Cu and CPG showed that after GOxmodification, CPG still has characteristic diffraction peaks corresponding to CuS (Figure 2C). Zeta potential can reflect the interaction between nanostructures and is a critical property of nanomaterials. The zeta potentials of CuS, CP, and CPG were −42 mV, −18.5 mV, and −11.2 mV, respectively (Figure 2D); surface modification leads to an increase in potential.

As CPG is capable of catalyzing H_2_O_2_ to generate hydroxyl radicals, which can degrade methyl violet (CAS), we reacted different nanoparticles with H_2_O_2_ and discovered that the absorption peaks of CAS in the CPG group were significantly lower (Figure 2E), indicating the successful generation of hydroxyl radicals.

We evaluated the temperature of the nano-hydrate solutions by determining the temperature rise of CPG at various concentrations (100 µg/mL, 200 µg/mL, and 300 µg/mL) in ultrapure water under 808 nm near-infrared (NIR) irradiation for 10 min. The results indicate that CPG (100 µg/mL) could be heated to 46 °C within 10 min (Figure 2F), and the temperature rose more rapidly at a higher power (Figure S1). Moreover, we detected no significant changes in the photothermal conversion performance of the nanoparticles over three cycles (Figure 2G), and we have constructed the cooling curve of nanoparticles (Figure S2), suggesting good thermal stability. In conclusion, the nanoparticles display excellent photothermal conversion efficiency and thermal stability.

### 2.2. Preparation and Characterization of Hydrogels

**Figure 3:**
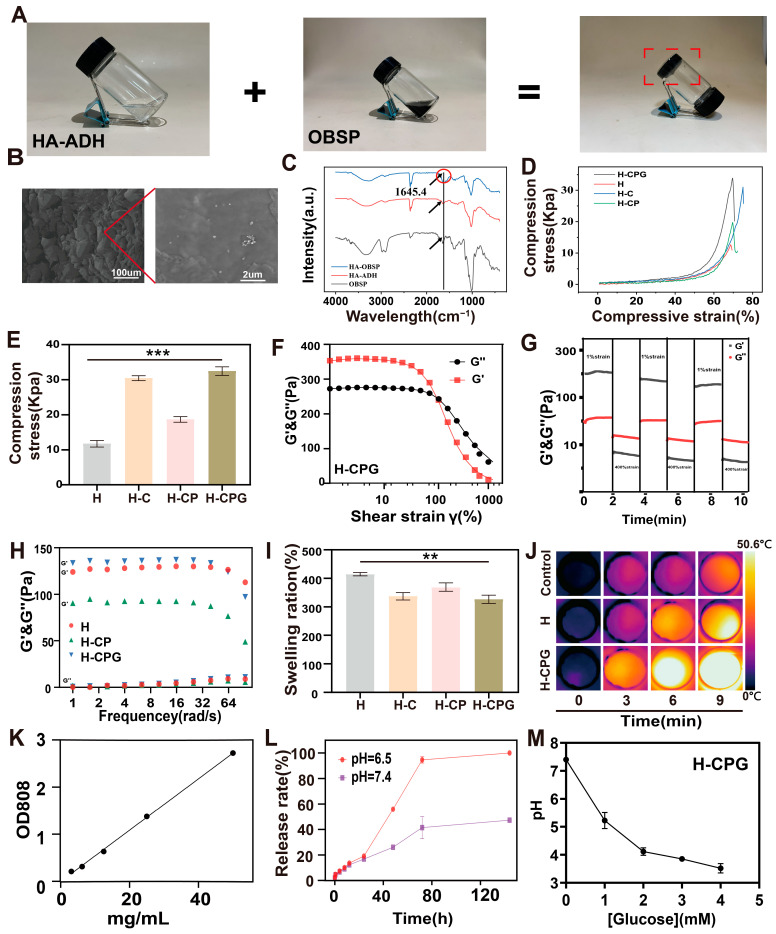
Morphology, degradation properties, mechanical properties, and photothermal properties of H-CPG hydrogels: (**A**) Optical images of hydrogels. The two solutions of HA-ADH and OBSP were mixed in the same volume. (**B**) SEM plots of the hydrogels of H-CPG. (**C**) FTIR plots of H-CPG, HA-ADH, OBSP. (**D**) Tensile stress–strain curves of the hydrogels of H, H-C, H-CP, H-CPG. (**E**) H, H-C, H-CP, H-CPG hydrogels of maximum withstand pressure plots. (**F**) H-CPG strain scans showing gel–sol transition points. (**G**) G′ and G″ change curves of H-CPG hydrogel at alternating high strain (400%) and low strain (1%). (**H**) Oscillation frequencies (1–100 rad/s) of scanned H-CPG hydrogels at 37 °C and 1 Hz. (**I**) H, H-C, H-CP, H-CPG hydrogels swelling properties in PBS at 37 °C. (**J**) Photothermal images of PBS, H, and H-CPG at 10 min. (**K**) Standard curve of CuS@PDA@GOx. (**L**) CPG release profiles of H-CPG at (pH = 7.4) and (pH = 6.5). (**M**) Degradation of glucose by H-CPG hydrogels. (Mean ± SD (*n* = 3) ). ** *p* < 0.01, and *** *p* < 0.001.

The carboxyl terminus of hyaluronic acid was grafted with adipic dihydrazide to acquire amino groups. The carboxyl end of the oxidized hyaluronic acid polysaccharide was oxidized to yield aldehyde groups. Subsequently, through the dynamic Schiff base bonding approach, this hydrogel was successfully fabricated. We mixed 1% HA-ADH and 1% OBSP to dissolve CPG (OBSP-CPG) at a 1:1 volume ratio. Upon inversion, they did not flow down within 5 min, signifying the successful formation of hydrogels (Figure 3A). To better observe the three-dimensional structure of the hydrogels, SEM images of the H-CPG hydrogels were prepared, revealing a homogeneous three-dimensional mesh structure (Figure 3B). Additionally, the presence of CPG nanoparticles was conspicuously observed on the surface of the hydrogel. The Fourier-transform infrared (FTIR) spectrogram of the HA-OBSP hydrogel exhibited a C=O stretching vibrational peak at 1720.4 cm^−1^ (Figure 3C), indicating the successful formation of an imine structure. We investigated the compressive properties of hydrogels before and after the addition of nanoparticles. The hydrogel, after loading CPG, could withstand a pressure of 30 ± 2 kPa (Figure 3D,E), suggesting that the incorporation of nanoparticles can enhance the compressive capacity of hydrogels. Furthermore, we employed rheological methods to determine the energy storage modulus (G′) and loss modulus (G″) of the hydrogels. The intersection of G′ and G″ of the H-CPG hydrogels was 212% (Figure 3F), signifying the shear-thinning point of the hydrogels and the subsequent structural collapse, mainly due to reversible forces. In this study, hydrogels were ultimately obtained by crosslinking HA-ADH and OBSP with the aid of dynamic Schiff bases and hydrogen bonding. Under high-shear conditions, the dynamic bonding of the hydrogels was disrupted, and they were transformed from the original solid to a fluid-like state, which was restored when the shear force was removed. It was demonstrated by the alternating strain tests of 1% and 400% that when the shear strain was increased from 1% to 400%, the G″ of the H-CPG hydrogels was higher than G′ (Figure 3G), and the hydrogel structure collapsed; when the strain was shifted from 400% to 1%, the G′ of the hydrogel was higher than G″, indicating that the hydrogel returned to the gel state. In the case of constant-strain frequency scanning, the G″ of each group of hydrogels was still higher than G′ (Figure 3H), indicating the structural stability of the hydrogels. The viscosity of all hydrogels decreased with an increasing shear rate, as indicated by the shear-rate scanning curves (Figure S3). The initial linear region is referred to as the linear viscoelastic region. A linear viscoelastic material responds instantaneously and elastically to stress or strain within its linear viscoelastic zone. This indicates that there is no permanent deformation of the material after the removal of tension or strain, confirming its shear-thinning.

Swelling properties are a fundamental property of hydrogels. We investigated the swelling capacity of these composite hydrogels. The swelling rates of H, H-C, H-CP, and H-CPG were 412 ± 5%, 335 ± 10%, 374 ± 5%, and 312 ± 7% (Figure 3I), which indicates that the CPG doped composite hydrogel has lower swelling properties compared with the blank hydrogel. Such morphologies imply more cross linkages within the gel matrix, resulting in decreased porosity. The reduction in porosity significantly hinders the water uptake behavior, namely, swelling. Additionally, we investigated the photothermal properties of the H-CPG hydrogel. The results show that the hydrogel can be heated to 45.5 °C within 9 min (Figure 3J).

The release of drugs in the tumor micro-environment is of great significance for preventing tumor recurrence. By utilizing the standard curve of CPG and calculating its release rate, we discovered that the H-CPG hydrogel releases less at pH 7.4 and can release more rapidly in the tumor micro-environment with an acidic pH of 6.5 (Figure 3L), facilitating rapid tumor killing. To test the glucose-degradation behavior of this hydrogel, we dispersed H-CPG in glucose solutions with different concentration gradients (0–4 mM) and observed a significant decrease in pH after 48 h (Figure 3M), which indicated the production of gluconic acid.

### 2.3. In Vitro Anti-Tumor Evaluation

**Figure 4:**
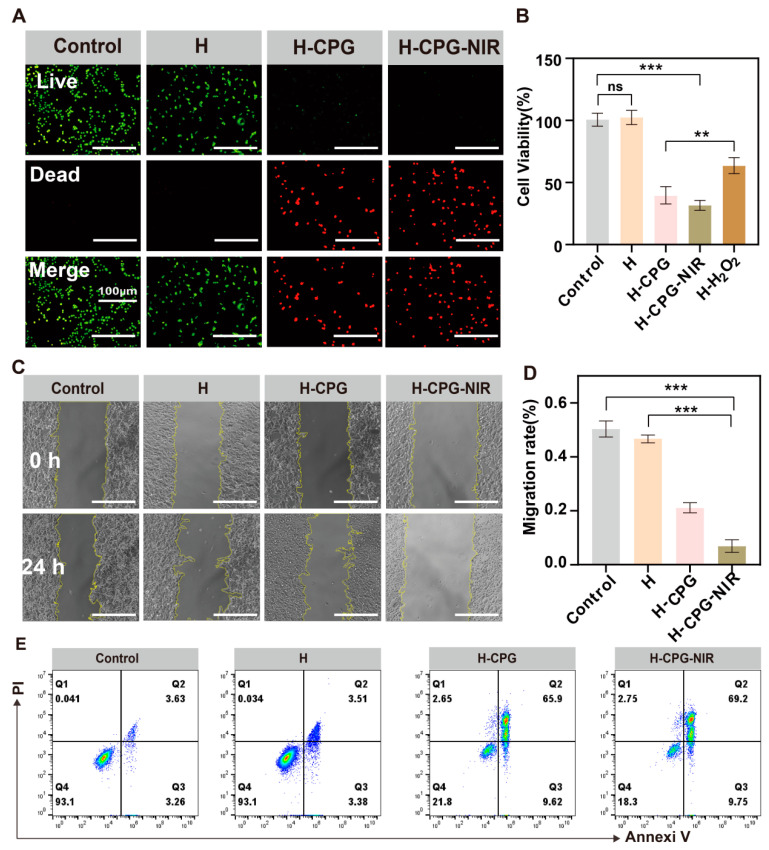
In vitro antitumor activity of hydrogels: (**A**) Live/dead staining images of B16F10 cells after various treatments. Scale bar: 100 µm. (**B**) Cell viability of B16F10 cells after different materials treatments. (**C**) Migration images of B16F10 cells after different materials treatments. Scale bar: 100 µm. (**D**) Visualized mobility of B16F10 cells after different materials treatments. (**E**) Flow-through apoptosis images of B16F10 cells after different materials treatments. (Mean ± SD (*n* = 3)). ns indicates no significant difference. ** *p* < 0.01, and *** *p* < 0.001.

An experiment was performed to investigate the effect of different treatments on the viability of B16F10 cells via the MTT assay. The average cell viabilities in the Control, H, H-CPG, H-CPG-NIR, and H-H_2_O_2_ groups were 100.5 ± 2%, 101 ± 2%, 40.1 ± 5%, 21.8 ± 3%, and 58.1 ± 6%, respectively (Figure 4B). These results implied that the H-CPG-NIR group demonstrated a remarkable ability to kill tumor cells, while the H-H_2_O_2_ group alone had a higher cell survival rate compared to the H-CPG group. The utilization of oxidative stress (ROS) did not result in significant cell death, presumably due to the high energy demand of tumor cells for rapid proliferation and the limited glucose availability that severely impeded their rapid growth. The survival rate of tumor cells in the H-CPG-NIR group was lower than that in the H-CPG group, suggesting that the combined photothermal therapy enhanced the mortality of tumor cells. This is because mild photothermal therapy not only directly inhibits the proliferation of tumor cells but also amplifies the effects of starvation therapy. Additionally, live/dead cell staining (AO/EB) corroborated these results.

We also explored the inhibitory effect of CPG on tumor cell migration by means of a cell scratch assay. The cell migration rate of H-CPG-NIR was decreased to 0.05%, indicating that CPG has a more prominent anti-tumor migration effect under light conditions (Figure 4D). Tumor cells treated with H-CPG-NIR showed a significant reduction in migration ability, which further emphasizes the potential of this treatment in preventing tumor metastasis.

In addition, we explored the mechanisms underlying cell death. The results of flow cytometry indicated that the percentages of apoptotic cells in the Control, H, H-CPG, and H-CPG-NIR groups were 3.85 ± 1%, 3.51 ± 1.5%, 65.9 ± 6%, and 77.1 ± 7%, respectively (Figure 4E). This implies that H-CPG-NIR induces cancer cell death more via apoptosis rather than cell necrosis.

The allowable hemolysis rate of biomaterials is lower than 5%. The hemolysis rate of all hydrogel groups was less than 5% (Figure S4), suggesting that the hydrogels possess good blood compatibility and do not cause hemolysis. The biocompatibility test demonstrated that the composite hydrogel does not exert obvious toxicity on normal cells (Figure S5).

### 2.4. Exploring the Mechanisms of Cancer Cell Death

**Figure 5:**
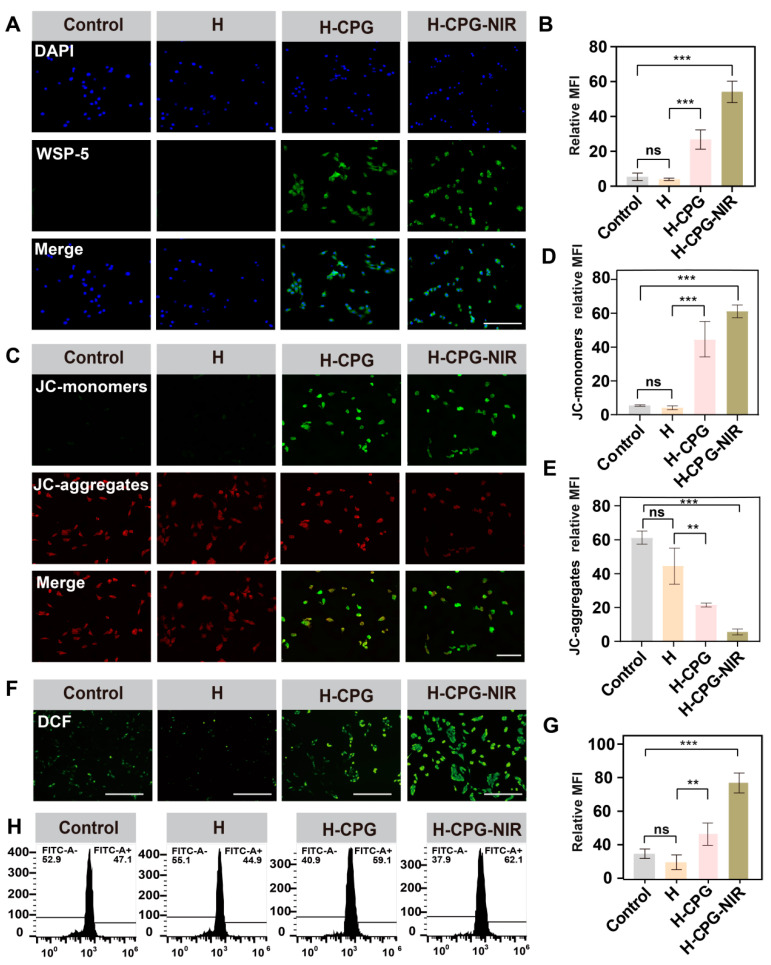
(**A**) Intracellular H_2_S levels after different treatments using WSP-5 as a probe. Scale bar: 200 µm. (**B**) Mean fluorescence intensity of H_2_S corresponding to A. (**C**) Microscopic measurements of intracellular mitochondrial damage by JC-1 probe. Scale bar: 100 µm. (**D**) Mean fluorescence intensity of JC-monomers corresponding to Figure B. (**E**) Mean fluorescence intensity of JC-aggregates corresponding to Figure B. (**F**) By staining with Dichloride hydro fluorescein diacetate (DCFH-DA) probe to assess the ROS levels of B16F10 cells after different materials treatments. Scale bar: 200 µm. (**G**) Fluorescence intensity of the DCFH-DA probe corresponding to Figure E. (**H**) ROS levels of B16F10 cells were analyzed by flow cytometry. (Mean ± SD (*n* = 3)), ns indicates no significant difference. ** *p* < 0.01, and *** *p* < 0.001.

To understand the mechanisms of cancer cell death, we investigated the indicators related to CDT. In this study, CPG can react with intracellular glucose and O_2_ to generate H_2_O_2_ and gluconic acid, and the concentration of H_2_O_2_ is significantly increased with the increase in CPG concentration (Figure S6). The H2S-specific fluorescent probe wsp-5 was utilized to detect the release of H_2_S from the cells. No significant fluorescence was observed in both the Control and H groups, suggesting the non-detection of H_2_S (Figure 5A). In the H-CPG and H-CPG-NIR groups, the cells presented high levels of H_2_S, and the fluorescence was stronger in the 808 nm NIR group, which was attributed to the fact that light accelerates the release of H_2_S. Mitochondria play a crucial role in tumorigenesis. The mitochondrial membrane potential (MMP) JC-1 probe is a commonly used indicator. JC-1 aggregates fluorescent red in healthy mitochondria, while JC-1 monomers emit fluorescent green in abnormal mitochondria depolarized by MMP. The strongest green fluorescence was observed in the H-CPG-NIR group, suggesting that CPG causes the most significant mitochondrial dysfunction by releasing H_2_S (Figure 5C).

H_2_O_2_ is capable of catalyzing the generation of hydroxyl radicals (•OH) from Cu^2+^ with the assistance of the Fenton reaction, thereby inducing mitochondrial ROS. We explored the level of oxidative stress in the cells by using DCFH-DA. The group to which CPG was added exhibited a distinct green fluorescence (Figure 5E), while the other groups had a relatively weaker fluorescence level. Furthermore, as further verified by flow cytometry, the percentages of positive cells in the Control group, the H group, the H-CPG group, and the H-CPG-NIR group were 47.1 ± 0.7%, 44.1 ± 1.5%, 59.1 ± 3%, and 62.1 ± 2.1%, respectively (Figure 5G), indicating that the CPG group had a higher degree of fluorescence binding. The deterioration of the intracellular oxidative status inevitably alters the concentration of GSH. Compared with the other groups, the H-CPG group had the lowest level of intracellular GSH (Figure S7).

Intracellular ATP is a product of energy production and provides cells with nutrients to carry out all life activities. Both H-CPG and H-CPG-NIR decreased ATP production (Figure S8), suggesting that GOx-mediated starvation therapies can competitively deprive cells of intracellular glucose.

### 2.5. In Vitro Evaluation of Macrophage Polarization

**Figure 6:**
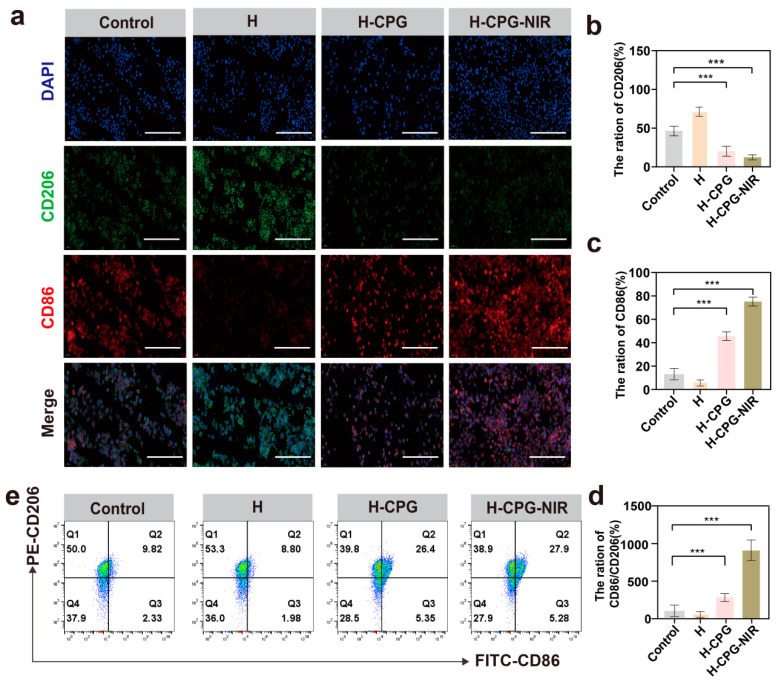
H-CPG-induced immunotherapy: (**a**) Immunofluorescence of TAM cells after different materials treatments. Scale bar: 100 µm. (**b**) Percentage of CD206 positive cells corresponding to Figure A. (**c**) Percentage of CD86 positive cells corresponding to Figure A. (**d**) Percentage of CD86/CD206 cells corresponding to Figure A. (**e**) Flow chart of TAMs cells after different materials treatments. (Mean ± SD (*n* = 3)). *** *p* < 0.001).

We investigated the effect of H-CPG hydrogel on macrophages, particularly M1 TAMs, as they have been shown to contribute to antitumor effects. We focused on the polarizing effect of H-CPG hydrogel on macrophages and selected two markers, CD86 and CD206, for our study. The results revealed that the relative fluorescence intensity of CD206 in the H-CPG and H-CPG-NIR groups was 23.2 ± 3% and 18.2 ± 1.5%, respectively (Figure 6a, Figure 6b), which was significantly lower than in the Control group. Additionally, the expression of CD86 was much higher in the H-CPG group and H-CPG-NIR group compared to the Control and H groups (Figure 6c, Figure 6d), respectively. Furthermore, flow cytometry results demonstrated that the percentage of CD86 in the Control, H, H-CPG, and H-CPG-NIR groups were 9.82±1.8%, 8.80±1.5%, 26.4±2.0%, and 27.9±3.4% (Figure 6e), with the CD86 expression being increased in the H-CPG-NIR group. In conclusion, our findings suggest that H-CPG-NIR can reverse the immunosuppressive state of the tumor microenvironment by remodeling M2 TAMs.

### 2.6. Evaluation of Antimicrobial Properties

**Figure 7:**
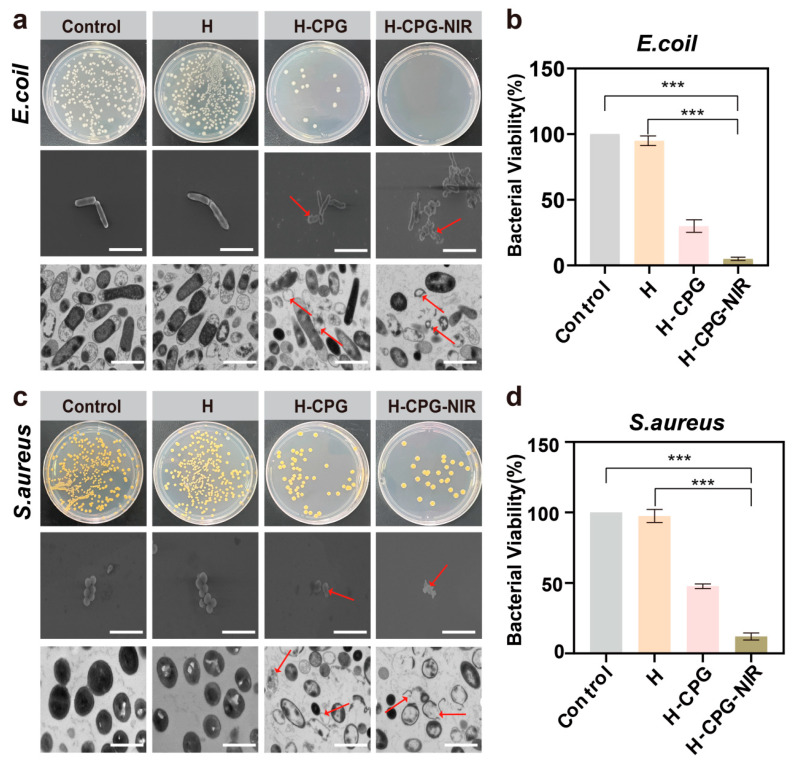
Evaluation of the antimicrobial effect: (**a**) Antimicrobial effect of different materials against *E. Coil*. The arrow points to the damaged bacterial structure. Scale bar: 2 µm. (**b**) Relative survival of *E. coli* corresponding to Figure a. (**c**) Antimicrobial effect of different materials against *S. aureus*. Scale bar: 2 µm. (**d**) Relative survival of *S. aureus* corresponding to Figure d. (Mean ± SD (n = 3). *** *p* < 0.001).

The management of wound care confronts challenges in preventing wound infection and maintaining the appropriate moisture level around the wound. When assessing the antimicrobial performance through the direct contact method, the survival rates of *E. coli* for H-CPG and H-CPG-NIR were 31.4 ± 6.8% and 7.6 ± 1.1%, respectively (Figure 7b), and it was evident that the cell membranes of the bacteria had been ruptured (Figure 7a). On the other hand, the survival rates of S. aureus for H-CPG and H-CPG-NIR were 42.6 ± 5.2% and 18.6 ± 4.1%, respectively (Figure 7d), and it was clear that both the cell membrane and cell wall of the bacteria had been ruptured (Figure 7c). The survival rates of the bacteria in the other groups were significantly high. This is attributed to the photothermal properties of the H- CPG hydrogel, which can convert light energy into heat, increasing the local wound temperature to kill bacteria. Additionally, CPG can catalyze the production of •OH from H_2_O_2_ to disrupt the bacterial structure and cause bacterial death. Therefore, combining nano-kinetic therapy with photothermal therapy represents a promising antimicrobial strategy.

### 2.7. In Vivo Evaluation of Anti-Tumor

**Figure 8:**
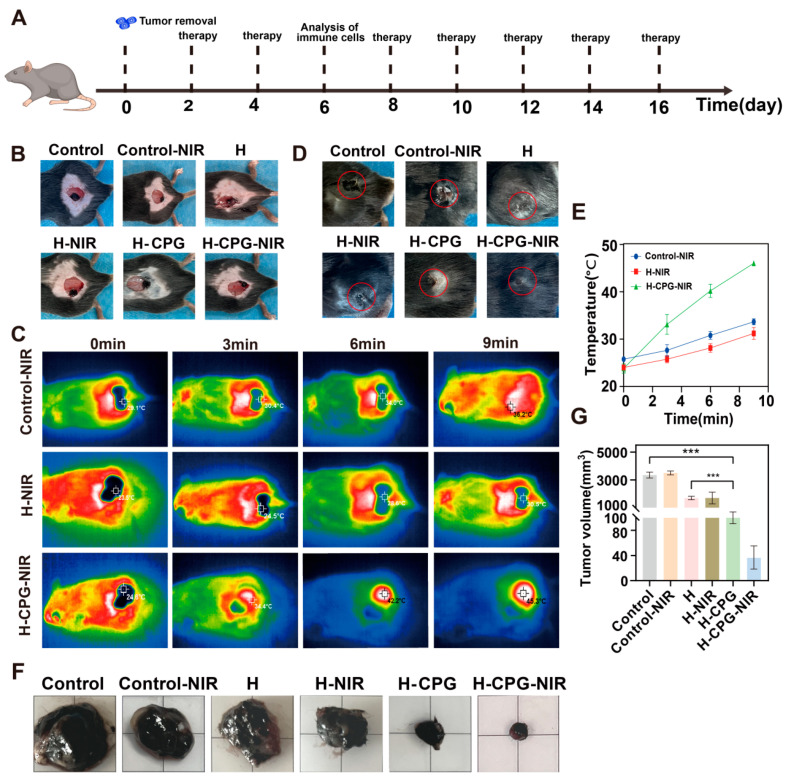
(**A**) Postoperative treatment protocols for tumors in mice in vivo. (**B**) Postoperative tumor resection model. (**C**) Thermograms of Control, H, and H-CPG group mice after 10 min of irradiation with 808 nm near-infrared radiation (1.0 W/cm^2^). (**D**) Tumor and wound repair in mice after 18 days of treatment, The red circle part represents the tumor size and wound distribution after 18 days of treatment. (**E**) Temperature distribution range corresponding to Figure C. (**F**) Tumor size in mice after 18 days of treatment. (**G**) Corresponds to (**F**) the size of the tumor volume. (Mean ± SD (*n* = 5)). *** *p* < 0.001.

We investigated the role of the composite hydrogel in inhibiting tumor recurrence after surgery. The treatment protocol is depicted in Figure 8A. The Control, H, and H-CPG hydrogels, along with the NIR groups exposed to 808 nm (1.0 w/cm^2^) NIR for 10 min, were utilized to treat tumor-induced wounds. The temperature of the tumor area in the H-CPG-NIR group was maintained at approximately 46.3 °C, whereas that of the H-NIR and the Control NIR groups was only 32 °C (Figure 8C). It was also observed that the mice in the H-CPG group had the most rapid increase in back temperature (Figure 8E). The tumors of mice in the Control, H, Control-NIR, and H-NIR groups were significantly enlarged (Figure 8F). In terms of the removed tumor volume, the tumor volume of the H-CPG-NIR group was significantly smaller than that of the other groups (Figure 8G). Virtually no tumor recurrence was detected in the H-CPG group. These results confirmed that H-CPG-NIR demonstrated excellent efficacy in inhibiting tumor recurrence.

**Figure 9:**
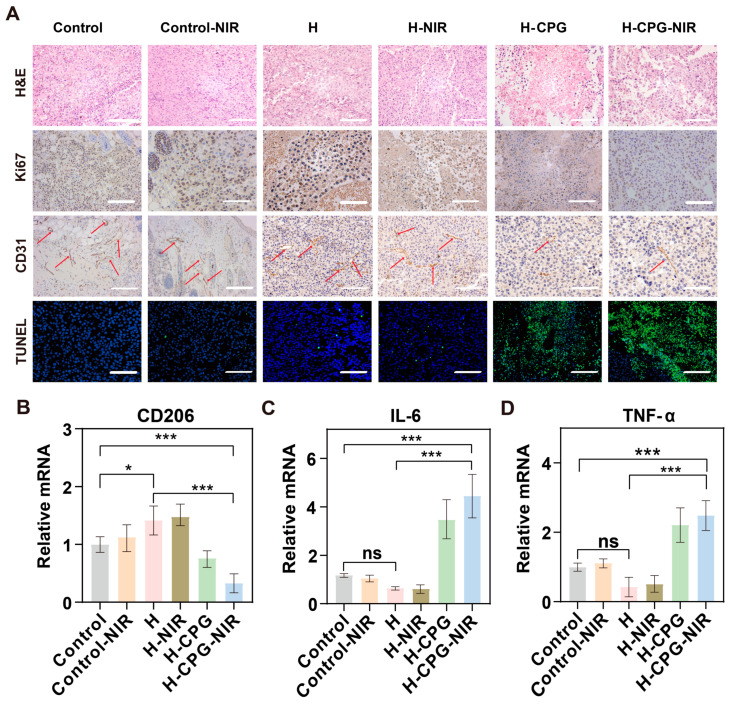
(**A**) Photographs associated with histological analysis of tumor cells after 18 days of treatment. The red arrow represents the tumor vascular marker. Scale bar: 200 µm. (**B**) Percentage of CD206 immune cells after treatment with different drugs.(**C**) Percentage of IL-6 immune cells after treatment with different drugs. (**D**) Percentage of TNF-α immune cells after treatment with different drugs. (Mean ± SD (*n* = 5)), ns indicates no significant difference. * *p* < 0.05, and *** *p* < 0.001.

Moreover, the H&E staining images of the tumor suggested that the tumor cells within the H-CPG-NIR and H-CPG groups presented a folded appearance and held a considerable number of necrotic cells, in contrast to the other groups where almost no necrotic areas were observed among the tumor cells (Figure 9A). The TUNEL staining images also suggested that a greater number of apoptotic cells were present in the H-CPG-NIR group (Figure 9A). Ki67 is expressed in proliferating cells. The expression level of Ki67 was significantly lower in H-CPG and H-CPG-NIR, while it was conspicuously higher in the Control, H, Control-NIR, and H-NIR groups (Figure 9A). Tumorigenesis and metastasis are contingent upon tumor angiogenesis, as the tumor vasculature provides the conditions for tumor recurrence and escape. Consequently, we investigated the angiogenesis marker (CD31). CD31 was barely expressed in the H-CPG and H-CPG-NIR groups (Figure 9A). In summary, H-CPG has the ability to inhibit tumor angiogenesis, thereby preventing tumor cell metastasis to a certain extent.

On the 18th day after the operation, sections of major organs were collected and stained with H&E. The results showed that there were no obvious pathological abnormalities observed in major organs such as the heart, liver, spleen, lungs, and kidneys in all groups (Figure S9), thus proving the biosafety of the multifunctional hydrogels.

### 2.8. Evaluation of the Immune Response In Vivo

Based on their nature and function, macrophages can be classified into two main types: M1 and M2. M1, or pro-inflammatory macrophages, are primarily involved in necrosis, microbial clearance, and tumor clearance by secreting pro-inflammatory cytokines and chemokines. The remodeling of TAMs from M2 to M1 can stimulate the immune system to combat tumor cells. Consequently, we determined the expression of several relevant mRNAs in tumor cells through RT-qPCR. The expression of CD206 in the H -CPG and H-CPG-NIR groups was significantly lower than that in the Control group, with values of 0.75 ± 0.1% and 0.52 ± 0.1%, respectively (Figure 9B). The relative expression of IL-6 and TNF-α in the H-CPG group and the H-CPG-NIR group was 3-fold and 1.8-fold higher than that in the Control group, respectively (Figure 9B), which directly indicates that the addition of CPG regulates the remodeling of M2 TAMs into M1 TAMs. We selected CRT and HMGB1 for staining and analyzed that this hydrogel could induce ICD effects (Figure S10). In the H-CPG group, CRT was increased and HMGB1 expression was decreased, which is an important “eat-me signal” in the immunogenic cell death (ICD) cascade. As expected, the hydrogel in the H-CPG group demonstrated a strong anti-tumor immune response. In conclusion, the combination of starvation therapy and chemokinetic therapy can effectively enhance the ICD effect.

## 3. Methods and Materials

### 3.1. Materials

BSP (M_W_ = 10 KDa) was obtained from Xi’an Ben yin Biological Co. Ltd. (Xi’an, China). Copper chloride dihydrate (CuCl_2_-2H_2_O, 99%), HA (M_W_ = 10 KDa), ADH (99%), GOx (from Aspergillus Niger, 100 U/mg), sodium sulfide (Na_2_S·9H_2_O, 99%), polyvinylpyrrolidone (PVP K30, MW = 40,000, 99%), sodium periodate (99.8%), N_2_H_4_-H_2_O (99%), 1-Ethyl-(3-dimethylaminopropyl)carbodiimide hydrochloride (EDC, 98%), nhydroxy succinimide (NHS, 98%), dopamine hydrochloride (98%) were purchased from Shanghai Aladdin Co. (Shanghai, China). Roswell Park Memorial Institute medium 1640 (RPMI 1640) was purchased from Gibo Ltd. (Reading, MA, USA). All other reagents used in the experiments were of analytical grade and did not require further purification.

### 3.2. Preparation of CuS Nanoparticles and CuS@PDA Nanoparticles

Hollow copper CuS were prepared according to previously reported literature and used as templates [24]. CuS@PDA nanoparticles obtained by surface loading of CuS with polydopamine. Detailed information can be found in the Supporting Information.

### 3.3. Preparation of CuS@PDA@GOx Nanoparticles

CuS@PDA@GOx was obtained by adding GOx (5 mg) to a solution containing CuS@PDA (10 mg) with stirring. To determine the loading capacity of GOx on CuS@PDA@GOx NPs, the unbound proteins remaining after affix synthesis and isolation were calculated using the Bradford assay. Detailed information can be found in the Supporting Information.

### 3.4. Preparation of OBSP 

Preparation of aldolase BSP (OBSP) by oxidation of BSP using sodium periodate as oxidant [25]. Detailed information can be found in the Supporting Information.

### 3.5. Preparation of HA-ADH

Modification of the carboxyl group of HA using the amino group of ADH to obtain HA-ADH [26]. Detailed information can be found in the Supporting Information.

### 3.6. Preparation of Multifunctional Hydrogels

The prepared CuS, CuS@PDA (CP), and CuS@PDA@GOx (CPG) nanoparticles were dispersed in OBSP (1%) solution and ultrasonically mixed, respectively; HA-ADH (1%) was then added to each of these mixed solutions in a 1:1 volume ratio, respectively; after mixing in a vortex oscillator, it was inverted to observe the flow. The hydrogels obtained were named hydrogel-CuS (H-C), hydrogel-CuS@PDA (H-CP), hydrogel- CuS@PDA@GOx (H-CPG), the hydrogels of undoped nanoparticles were named H.

### 3.7. Determination of Photothermal Properties of Nanoparticles

To comprehensively explore the photothermal performance, CPG nanoparticle solutions with concentrations of 100 µg/mL, 200 µg/mL, and 300 µg/mL were meticulously configured. Subsequently, these nanoparticle solutions were irradiated using an 808 nm near-infrared (NIR) emitter at a power density of 1.0 W/cm^2^, and the temperature was meticulously recorded at intervals of 30 s. The magnitudes of temperature changes in the solutions over a 10 min period were accurately documented. In addition, the photothermal efficiency of CPG at a concentration of 100 µg/mL was evaluated at different power densities of 1 W/cm^2^, 1.5 W/cm^2^, and 2 W/cm^2^. Moreover, three cycles of heating and cooling were conducted for CPG (100 μg/mL) under 808 nm NIR irradiation at a power density of 1.0 W/cm^2^ to assess the stability of the photothermal properties of the nanoparticles.

### 3.8. Determination of Physicochemical Properties of Hydrogels

The physicochemical properties of hydrogels were studied by hydrogel morphology, swelling properties, mechanical properties, and hemolysis experiments. Detailed information can be found in the Supporting Information.

### 3.9. Photothermal Properties of Hydrogels

H-CPG and H hydrogels were placed in a 24-well plate and the hydrogel was irradiated using an 808 nm NIR emitter at 1.0 W/cm^2^, and the thermograms were recorded every 3 min with a thermal imager. Thermal imaging of the hydrogel was recorded over a period of 10 min.

### 3.10. CPG Release Analysis

Firstly, the standard curve was obtained by assessing the absorbance of CPG with equal-gradient concentrations. The H-CPG hydrogels were incubated in PBS buffer solutions at pH = 6.5 and pH = 7.4 for 72 h in a constant-temperature water-bath shaker at 37 °C. During this period, 1 mL of the slow-release solution was aspirated at regular intervals, and then 1 mL of fresh PBS buffer was added back to continue the experiment. The buffer solution was precipitated, and the absorbances under the two solutions were determined separately. By comparing with the standard curve, the CPG content in its solution was obtained.

### 3.11. Decomposition Glucose Test

The 5 g H-CPG hydrogel (1 g/mL) was added to 4.5 mL of PBS (pH = 7.4) containing different concentrations of glucose (0–4 mM) and incubated for 48 h at 37 °C and gently shaken to measure its pH.

### 3.12. In Vitro Anti-Tumor Evaluation

The B16F10 density was adjusted to 1 × 10^5^ cells/mL, added to 96-well plates with a volume of 100 μL per well, and placed in a 37 °C incubator for 24 h. After 24 h, the extracted H (1 g/mL), H-CPG (1 g/mL), H-CPG-NIR (1 g/mL, 808 nm, 1.0 W/cm^2^, 10 min) medium was added, and the blank group was added with an equal volume of RPMI 1640 culture medium, and 5 sub-wells were set up for each concentration. After 24 h, a mixture of thiazolyl blue (MTT) and cell culture medium corresponding to a volume ratio of 1:2 was added to each well, and the incubation was continued for 4 h. The cell cultures were incubated for 2 h, then 150 μL of dimethyl sulfoxide (DMSO) was added and slowly shaken for 2 min, and the absorbance (OD) value corresponding to each well was measured at 490 nm using an enzyme meter, cell survival was calculated using Equation (1):

(1) $$\mathrm{Relative}\;\mathrm{cell}\;\mathrm{proliferation}\;\mathrm{rate}\;(\%)=\frac{{\mathrm{OD}}_{\mathrm{sample}}\times {\mathrm{OD}}_{\mathrm{blanks}}}{{\mathrm{OD}}_{\mathrm{Control}}\times {\mathrm{OD}}_{\mathrm{blanks}}}\;\times \mathrm{100}\%$$

Furthermore, all cell groups were stained using the live and dead cell staining method (AO/EB), and the growth condition of B16F10 cells cultured in the hydrogel extract was observed under a fluorescence microscope, followed by photographing and documentation. The aforementioned cell-culturing procedure was repeated, and 6-well plates were re-coated with a volume of 2 mL per well and placed in a 37 °C incubator. Once the cells grew to approximately 90% confluence, scratch assays were performed. Subsequently, H (1 g/mL), H-CPG (1 g/mL), and H-CPG-NIR (1 g/mL, 808 nm, 1.0 W/cm^2^, 10 min) were added after homogenization, while an equal volume of RPMI 1640 culture solution was added to the Control group. Incubation was continued for 24 h, and photographs were taken to record the state of the cells before and after migration. The mechanism of B16F10 cell death was investigated by apoptosis experiments. Detailed information can be found in the Supporting Information.

### 3.13. Regulation of Energy Metabolism In Vitro

The in vitro metabolic process of cancer cells was examined, and the mechanism of cancer cell death was explored through experiments such as hydrogen sulfide (H_2_S) content assay, reactive oxygen detection, reduced glutathione (GSH) content assay, adenosine triphosphate (ATP) content assay, and hydrogen peroxide (H_2_O_2_) content assay. Detailed information can be found in the Supporting Information.

### 3.14. Induced Macrophage Polarization

Mouse mononuclear-macrophage (RAW264.7) cells in the logarithmic growth phase were harvested and inoculated in 6-well plates at a density of 2 × 10^5^ cells/well, enabling them to grow until they reached confluence on the bottom of the well plates. One milliliter of 200 ng/mL interleukin-4 (IL-4) was added to each well to induce polarization of macrophages toward M2. Subsequently, the culture media of the extracted H (1 g/mL), H-CPG (1 g/mL), and H-CPG-NIR (1 g/mL, 808 nm, 1.0 W/cm^2^, 5 min) were added respectively, along with a Dulbecco’s modified Eagle medium (DMEM) blank Control group. The cells were then incubated in the incubator for 6 h. Subsequently, the effects of different extracts on the secretion of cluster of differentiation 86 (CD86; Proteintech Group (Wuhan, China), Cat No. 26903-1-AP) and cluster of differentiation 206 (CD206; Proteintech Group, Cat No. 60143-1-Ig) in RAW264.7 were investigated using an immunofluorescence assay, and the secretion of CD86 and CD206 was also quantified by flow cytometry.

### 3.15. Bacteriostatic Tests

We evaluated the antimicrobial function of the hydrogels by direct contact method. Detailed information can be found in the Supporting Information.

### 3.16. Construction and Treatment of Melanoma Wounds

All animal experiments were conducted in accordance with the National Research Council’s Guide for the Care and Use of Laboratory Animals and approved by the Animal Ethics Committee of Northwestern University, Shaanxi, China (NWU-AWC-20210810R). Melanoma modeling in 6- to 8-week-old C57BL/6 male mice by subcutaneous injection of 1 mL of B16F10 cells (2 × 10^7^ cells/mL). Incomplete tumor resection was performed when the tumor volume reached approximately 200 mm^3^. Divide 42 mice into 7 mice per group for the Control group (simply wrapped in non-medicated gauze), Control-NIR group (simply wrapped in non-medicated gauze), H group, H-NIR group, H-CPG group, and H-CPG-NIR group. Record the size and final average weight of the recurrent tumors after 18 days of treatment.

Tumor tissues were collected after treatment and hematoxylin and eosin staining (H&E), tumor vascular markers (CD31, (Proteintech Group, Cat No. 19003-1-AP)), protein phosphatase 1 (Ki67, (Proteintech Group, Cat No. 28074-1-AP)), and tdT-mediated dUTP nick-end labeling (TUNEL, (Proteintech Group, Cat No. PF00006)) were measured in tumor tissues.

### 3.17. Assessment of the Immune Response In Vivo

The mice were euthanized to collect tumor tissue. The obtained tumor tissue was ground to obtain tissue homogenate. After these treatments, relative mRNA expression of interleukin 6 (IL-6, (Tsingke (Beijing, China))), tumor necrosis factor-alpha (TNF-α, (Tsingke)), glyceraldehyde-3-phosphate dehydrogenase (GAPDH, (Tsingke)), and CD206 (Tsingke) in diverse groups determined by Quantitative real-time RT-qPCR. The RT-qPCR sequences are shown in Table 1 below:

### 3.18. Statistical Analysis

All data values above are expressed as mean ± standard deviation (SD), differences between groups in the experiment were assessed by two-tailed *t*-tests using GraphPad Prism 6 software, where the statistical significance was * *p* < 0.05, ** *p* < 0.01, and *** *p* < 0.001, respectively.

## 4. Conclusions

In this study, we synthesized a multifunctional hydrogel loaded with CPG nanoparticles. CPG can trigger a sustained ROS cascade response by releasing GOx to generate gluconic acid and H_2_O_2_ and accelerating the formation of •OH. The continuous supply of ROS modulates the tumor micro-environment and augments the ICD-stimulated tumor immune responses. The simultaneous synergistic mild photothermal therapy maximizes the induction of apoptosis in cancer cells. Additionally, the multifunctional hydrogel can serve as a patch to cover the wound surface, separating it from external contact while the photothermal therapy eliminates the residual bacteria on the wound surface. The results demonstrated that this multifunctional hydrogel can inhibit tumor growth and recurrence, and this multifunctional composite hydrogel holds significant potential for clinical application in preventing recurrence after tumor surgery. Nevertheless, there are still several limitations. For example, the penetration ability of photothermal therapy is restricted, currently being confined to the treatment of skin tumors. Moreover, in the acidic tumor environment, the structural breakdown of the H-CPG hydrogel leads to the release of HA and BSP. The specific pathways of these two substances and the destinations of their degradation require further investigation. Additionally, this study did not conduct a comparison with commercially available drugs. A drug and hydrogel group should be established for a more comprehensive comparison.

## Figures and Tables

**Table 1:** RT-qPCR sequences.

Gene	Forward Primer	Reverse Primer
*GAPDH*	AGGTCGGTGTGAACGGATTTG	GGGGTCGTTGATGGCAACA
*TNF-* *α*	CCTGTAGCCCACGTCGTAG	GGGAGTAGACAAGGTACAACCC
*IL-6*	CTGCAAGAGACTTCCATCCAG	AGTGGTATAGACAGGTCTGTTGG
*CD206*	CTCTGTTCAGCTATTGGACGC	TGGCACTCCCAAACATAATTTGA

## Data Availability

Data is contained within the article and Supplementary Materials.

## Supplementary Materials

The following supporting information can be downloaded at: https://www.mdpi.com/article/10.3390/ijms252111465/s1.
