# Supplementary material for: Multifunctional Nanocomposite Hydrogel with Enhanced Chemodynamic Therapy and Starvation Therapy for Inhibiting Postoperative Tumor Recurrence
Source: Int J Mol Sci. 2024 Oct 25;25(21):11465. doi: 10.3390/ijms252111465 (PMC11547091; doi:10.3390/ijms252111465)
Supplement: Supplementary file 1 [file ijms-25-11465-s001.zip › ijms-3262537-supplementary.pdf]

Supporting Information

Multifunctional Nanocomposite Hydrogel with Enhanced Chemodynamic Therapy and Starvation Therapy for Inhibiting Postoperative Tumor Recurrence

Zeliang Li [a, b, c,1] and Xiaoxuan Ma [a, b, c,*]

[a] Engineering Research Center of Western Resource Innovation Medicine Green Manufacturing, Ministry of Education, School of Chemical Engineering, Northwest University, Xi'an, 710069, China

[b] Shaanxi Key Laboratory of Degradable Biomedical Materials and Shaanxi R&D Center of Biomaterials and Fermentation Engineering, School of Chemical Engineering, Northwest University, Xi'an, 710069, China

[c] Biotech. & Biomed. Research Institute, Northwest University, Xi'an, 710069, China

E-mail: xiaoxuanma@nwu.edu.cn

1. Experimental section

1.1 Preparation of CuS nanoparticles and CuS@PDA nanoparticles

Briefly, $CuCl_2$ -$2H_2O$ (17 mg) and PVP K30 (240 mg) were first dissolved in 100 mL of deionized water. Then N_2H_4-H_2O (29.4 μL) was added to the above reaction solution and stirred for 5 minutes. Na_2S (142 mg) was quickly added to the solution and kept at 60 °C for 2 hours. The reaction was then kept at 25 °C for 24 hours to synthesize CuS NPs, pure product was obtained by centrifugation at 10000 rpm for 10 minutes and washed three times with distilled water. Then CuS nanoparticles (60 mg) were dispersed in 30 mL of Tris-HCl buffer (10 mM, pH = 8.0) containing 7.5 mL of ethanol, 0.103 g dopamine (PDA) was poured in, and the solution was continuously stirred for 4 hours and centrifuged. The collected solid (CuS@PDA) was washed several times with ethanol.

1.2 Preparation of CuS@PDA@GOx nanoparticles

CuS@PDA (10 mg) was first dissolved in 10 mL of deionized water and sonicated, GOx (5 mg) was then dissolved in 10 mL of deionized water and mixed, after 24 hours of reaction at appropriate stirring rate, the synthesized CuS@PDA@GOx nanoparticles were separated by centrifugation at 10,000 rpm, then lyophilized and stored in a vacuum freeze dryer. The GOx loading was obtained by bradford protein concentration assay kit. Briefly, 5 μL of different concentrations of protein standards were added to the protein standard wells of a 96-well plate, 5 μL of CuS@PDA@GOx was added to the samples of the 96-well plate, and 250 μL of LG250 staining solution was added to each well, and the absorbance was measured between 560-610 nm.

Protein concentration was calculated from the standard curve.

1.3 Preparation of OBSP

Briefly, 4 g of BSP was dissolved in 200 mL of pure water, sodium periodate (glucose unit: $NaIO_4$ = 1: 0.2) was then slowly added and stirred for 24 hours at room temperature, protected from light, the reaction was quenched by stirring for 2 hours with equimolar ethylene glycol, dialysis was performed using dialysis bags with a cut-off molecular weight of 3500 Da, and finally dialyzed and freeze-dried.

1.4 Preparation of HA-ADH

Briefly, dissolve 1g of HA in 200 mL of deionized water, then EDC (four mole equivalents of the carboxyl group in HA) and NHS (four mole equivalents of the carboxyl group in HA) were added to activate the carboxyl group in HA, ADH (5 molar equivalents of carboxyl groups in HA) was added to the reaction mixture and stirred for another 24 hours at room temperature[34], the crude products were sequentially subjected to intensive dialysis with NaCl (0.1 M) and deionized water.

1.5 Characterization of hydrogels

The prepared hydrogels were completely frozen in an ultra-low temperature refrigerator at -80 ℃, and then the hydrogels were lyophilized using a vacuum freeze-dryer, the lyophilized hydrogel scaffolds were cut into small thin slices of uniform thickness, fixed on the carrier stage, and sprayed with gold. A scanning electron microscope (SEM) with 100× magnification

was used to observe the pore structure and distribution on the hydrogel surface and cut surface. The infrared spectrograms of HA-ADH, OBSP, and H-CPG hydrogels were determined by Fourier infrared spectroscopy, and the positions of the peaks were analyzed to determine the bonding states of the materials and to characterize the success of the synthesis of the hydrogels.

1.6 Mechanical properties of hydrogels

The rheological properties of hydrogels were characterize using a rheometer (MCR302). The experiments were conducted on parallel plates (20 mm diameter). Hydrogels were prepared in 24-well plates, and then measured by time-scan tests at a constant strain of 1% at 25 °C and a frequency of 1 Hz. Stability was measured using frequency sweep tests with hydrogels at 25 °C at a constant strain of 0.5% and frequency variations from 0.1 Hz to 100 Hz. Evaluation of hydrogels in compression by placing them on top of a universal test with a load cell, in the cyclic compression test, the hydrogel (cylindrical, 1 cm diameter, 1 cm thickness) was compressed for 1 cycle, the modulus data is calculated automatically by the INSTRON 5565 (Thermo Fisher) instrument.

1.7 Hydrogel swelling property test

The swelling behavior of the hydrogels were examined in phosphate buffer solution (PBS, pH=7.4). The mass of the lyophilized hydrogel was recorded as (M0). The freeze-dried hydrogels were immersed in PBS at 37 °C and weighed (M1) and recorded at predetermined time points after removing the surface solution of the hydrogels with filter paper. Define the swelling

ratio of the hydrogel using the following equation (1):

$$\text{Dissolution rate (\%)} = \frac{M_1 - M_0}{M_0} \times 100\% \qquad (1)$$

1.8 Blood compatibility

The blood compatibility of the materials was assessed in this study by determining their hemolysis rate. Briefly, 1 mL of fresh mouse blood was diluted with 25 mL of PBS. H, H-C, H-CP, and H-CPG (CPG, 100μg/mL) hydrogels were placed into test tubes containing 0.5 mL of physiological saline, followed by the addition of 0.5 mL of diluted blood, and incubated in a 37 °C water bath for 60 minutes. Deionized water and PBS without materials were used as positive and negative controls. All tubes were centrifuged at 2000 rpm for 10 minutes, and the absorbance at 540 nm of the supernatant.

1.9 Cytotoxicity assay

The L929 density was adjusted to 1×10^5 cells/mL, added to 96-well plates with a volume of 100 μL per well, and placed in a 37 °C incubator for 24 hours. After 24 hours, the extracted H (1 g/mL), H-CPG (1 g/mL), H-CPG-NIR (1 g/mL, 808 nm, 1.0 W/cm^2, 10 minutes) medium was added, and the blank group was added with an equal volume of RPMI 1640 culture medium, and 5 sub-wells were set up for each concentration. After 24 hours, a mixture of thiazolyl blue (MTT) and cell culture medium corresponding to a volume ratio of 1:2 was added to each well, and the incubation was continued for 4 hours. The cell cultures were incubated for 2 hours, then 150 μL of dimethyl sulfoxide (DMSO) was added and slowly shaken for 2 minutes, and the absorbance (OD) value corresponding to each well was measured at 490 nm using an enzyme meter.

2.0. Apoptosis experiments

B16F10 were cultured in RPMI 1640 medium supplemented with 10% (v/v) fetal bovine serum and 1% double antibody (100 U/mL penicillin and 100 μg/mL streptomycin). Cells with good growth status and cell density of 80% or more were taken, digested with trypsin, and collected by centrifugation, it was then suspended using an appropriate amount of RPMI 1640 culture medium to ensure homogeneity, take a small amount of the cell suspension and count it on a counting plate. According to the results obtained, the cell density was adjusted to 2×10^5 cells/mL, which was added to a 6-well plate with a volume of 2 mL per well and placed in 37 °C incubator. After 24 hours, the supernatant was discarded, and H, H-CPG, H-CPG-NIR (808 nm, 1.0 w/cm^2 for 10 minutes) were added after extraction, and Control was added to an equal volume of RPMI 1640 culture solution after 24 hours of incubation. Next, each group of media was collected into 15 mL centrifuge tubes and then rinsed with 1 mL of PBS to continue the collection into the corresponding centrifuge tubes, the adherent cells were digested with trypsin for 4 minutes, collected into the corresponding centrifuge tubes, continued to be resuspended with 1 mL of PBS and then collected by centrifugation, apoptosis was determined by flow cytometry after staining the samples with fluorescein isothiocyanate (FITC) and propidium iodide (PI) stains.

2.1. Measurement of oxidative stress levels

B16F10 were cultured in RPMI 1640 medium supplemented with 10% (v/v) fetal bovine serum and 1% double antibody (100 U/mL penicillin and 100 μg/mL streptomycin). Cells with good growth status and cell density of 80% or more were

taken, digested with trypsin, and collected by centrifugation, it was then suspended using an appropriate amount of RPMI 1640 culture medium to ensure homogeneity, take a small amount of the cell suspension and count it on a counting plate. According to the results obtained, the cell density was adjusted to 2×10^5 cells/mL, which was added to a 6-well plate with a volume of 2 mL per well and placed in a 37 °C incubator. After 24 hours, the supernatant was discarded, and H, H-CPG, H-CPG-NIR (808 nm, 1.0 w/cm^2 for 10 minutes) culture medium was added after extraction, and Control was added to an equal volume of RPMI 1640 culture solution after 24 hours of incubation. Next, serum-free medium containing dichloride hydro fluorescein diacetate (DCFH-DA, 100 μL, 10 μM) was added to petri dishes and incubated for 30 minutes to allow reaction with DCFH-DA to produce stable green fluorescence. The cells were rinsed three times with serum-free medium, then the staining was observed under a fluorescence microscope photographed and recorded.

2.2 H_2S generation experiment

B16F10 were cultured in RPMI 1640 medium supplemented with 10% (v/v) fetal bovine serum and 1% double antibody (100 U/mL penicillin and 100 μg/mL streptomycin), and the cells were incubated in a biochemistry incubator at 37 °C. Cells in good growth condition with cell density of 80% or more are taken, digested with trypsin, and collected by centrifugation. The cells were then suspended using an appropriate amount of RPMI 1640 culture medium to ensure homogeneity and counted. According to the results obtained, the cell density was adjusted to 2×10^5 cells/mL, and the cells were added into 6-well plates with a volume of 2 mL per well and placed in a

37 °C incubator. After 24 hours, the supernatant was discarded, and H, H-CPG, H-CPG-NIR (808 nm, 1.0 w/cm^2 for 10 minutes) were added after extraction, and Control was added to an equal volume of RPMI 1640 culture solution after 24 hours of incubation. After incubation with the addition of Washington state probe5 (WSP-5 probe) for 30 minutes, the cells were rinsed three times with PBS and then photographed for recording under an inverted microscope.

2.3 H_2O_2 generation experiments

B16F10 were cultured in RPMI 1640 medium supplemented with 10% (v/v) fetal bovine serum and 1% double antibody (100 U/mL penicillin and 100 μg/mL streptomycin), and the cells were incubated in a biochemistry incubator at 37 °C. Cells in good growth condition with cell density of 80% or more are taken, digested with trypsin, and collected by centrifugation. Cells were suspended with the appropriate amount of RPMI 1640 medium to ensure the same number and count. According to the results obtained, the cell density was adjusted to 2×10^5 cells/mL, and the cells were added into 6-well plates with a volume of 2 mL per well and placed in a 37 °C incubator. After 24 hours, the supernatant was discarded, after extraction, different concentration contents (0, 3, 6.25, 12.5, 25, 50 μg/mL) of H-CPG were added and incubated for 24 hours. The intracellular H_2O_2 production content was then measured using a hydrogen peroxide assay kit.

2.4 GSH reduction reaction experiment

B16F10 were cultured in RPMI 1640 medium supplemented with 10% (v/v) fetal bovine serum and 1% double antibody (100 U/mL penicillin and 100 μg/mL

streptomycin), and the cells were incubated in a biochemistry incubator at 37 °C. Cells in good growth condition with cell density of 80% or more are taken, digested with trypsin, and collected by centrifugation. The cells were then suspended using an appropriate amount of RPMI 1640 culture medium to ensure homogeneity and counted. According to the results obtained, the cell density was adjusted to 2×10^5 cells/mL, and the cells were added into 6-well plates with a volume of 2 mL per well and placed in a 37 °C incubator. After 24 hours, the supernatant was discarded, and H, H-CPG, H-CPG-NIR (808 nm, 1.0 w/cm^2 for 10 minutes.) were added after extraction, and Control was added to an equal volume of RPMI 1640 culture solution after 24 hours of incubation. GSH levels were then added using a reduced glutathione assay kit.

2.5 ATP reduction reaction experiment

B16F10 were cultured in RPMI 1640 medium supplemented with 10% (v/v) fetal bovine serum and 1% double antibody (100 U/mL penicillin and 100 μg/mL streptomycin), and the cells were incubated in a biochemistry incubator at 37 °C. Cells in good growth condition with cell density of 80 % or more are taken, digested with trypsin, and collected by centrifugation. The cells were then suspended using an appropriate amount of RPMI 1640 culture medium to ensure homogeneity and counted. According to the results obtained, the cell density was adjusted to 2×10^5 cells/mL, and the cells were added into 6-well plates with a volume of 2 mL per well and placed in a 37 °C incubator. After 24 hours, the supernatant was discarded, and H, H-CPG, H-CPG-NIR (808 nm, 1.0 w/cm^2 for 10 minutes.) were added after extraction, and Control was added to an equal volume of RPMI 1640 culture solution after 24 hours of incubation.

ATP levels were then added using a reduced glutathione assay kit.

2.6 Antimicrobial test

The antimicrobial efficacy of the composite hydrogels dressing was evaluated by the direct contact method, inoculation of 100 μL of logarithmic growth stage bacteria (Escherichia coli (*E. coli*), Staphylococcus aureus (*S. aureus*)) in sterile Luria-Bertani (LB) liquid medium. Control, sterilized H, sterilized H-CPG and sterilized H-CPG-NIR (808 nm, 1.0 w/cm^2 for 10 minutes) was immerse in LB medium, and then incubated at 37 °C for 24 hours. The OD values of the bacterial suspensions were obtained at 600 nm using an enzyme marker to characterize their viability and the bacterial survival was calculated using equation (2):

$$\text{Bacterial viability rate (\%)} = \frac{OD_{sampel} - OD_{blank}}{OD_{control} - OD_{blank}} \times 100\% \qquad (2)$$

At the same time, the bacterial suspension cultured for 24 hours was transferred to silicon wafers and fixed with 4% paraformaldehyde for 24 hours at 4 °C. Then dehydrated in 50%, 70%, 90% ethanol for 15 minutes and in 100% ethanol for 30 minutes, observation of its microstructure by SEM. Meanwhile, the above cultured bacterial suspension was centrifuge and separated, the supernatant was poured off, and added an electron microscope fixative to observe changes in bacterial structure under biological transmission electron microscopy.

Results

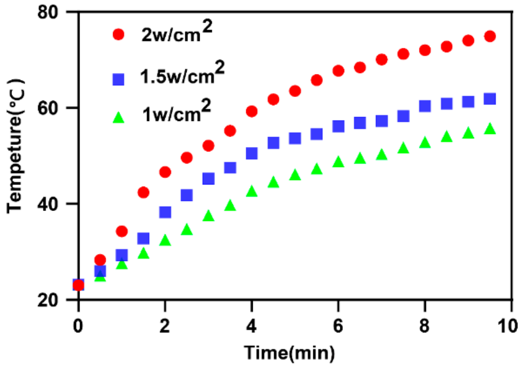

Figure S1. Photothermal heating curves of CPG (300 μg/mL) at 1.0 w/cm^2, 1.5 w/cm^2, 2 w/cm^2.

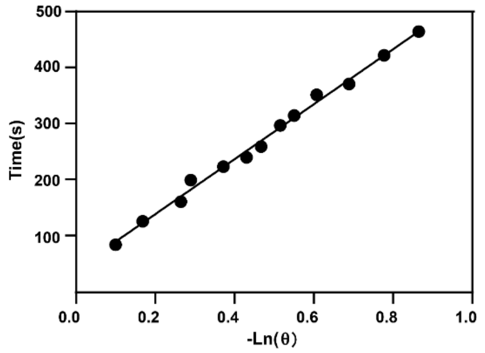

Figure S2. Cooling curves fitted under one cycle of photothermal for CPG (300 μg/mL).

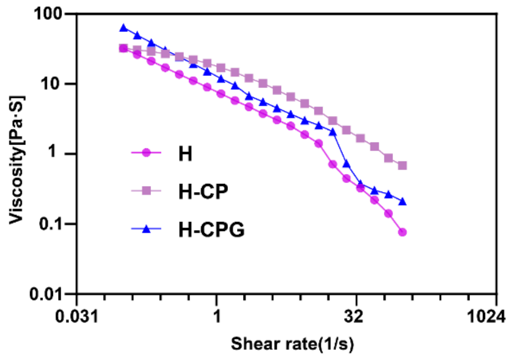

Figure S3. Shear rate scanning curves show that hydrogels are highly shear-thinning.

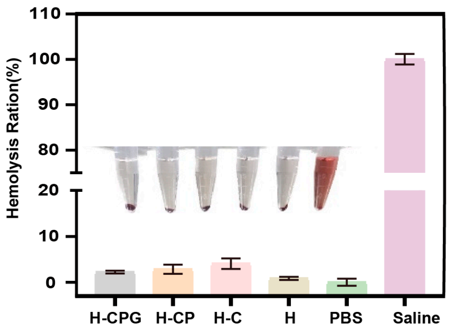

Figure S4. Hemolysis experiments in different groups.

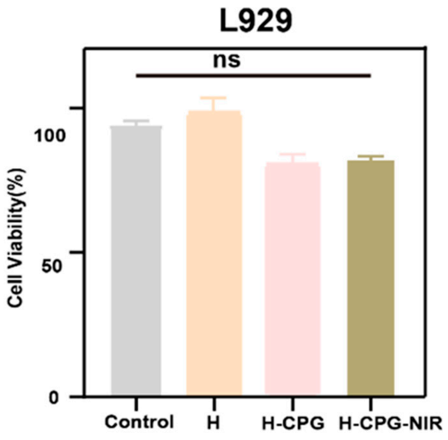

Figure S5. Cytotoxicity assay of H-CPG hydrogel on L929 cells.

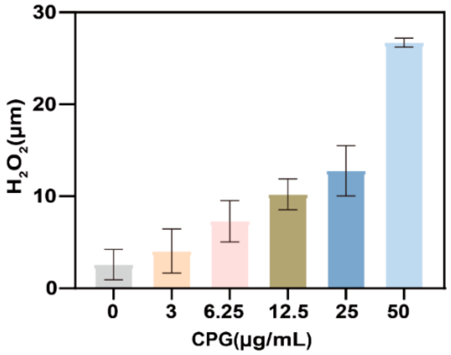

Figure S6. Changes of H_2O_2 content after treatment with different concentrations of H-CPG.

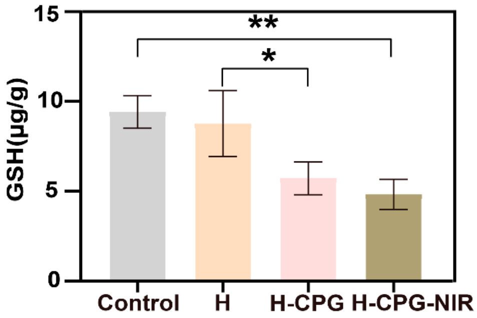

Figure S7. Changes in GSH content after different drug treatments.

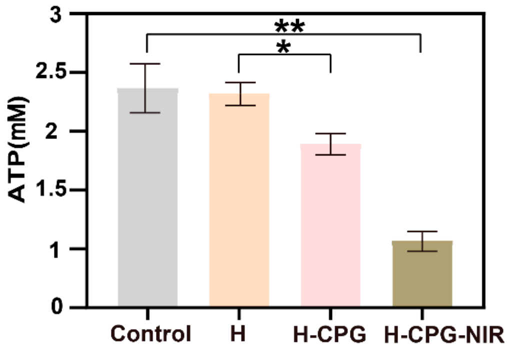

Figure S8. Changes in ATP content after different drug treatments.

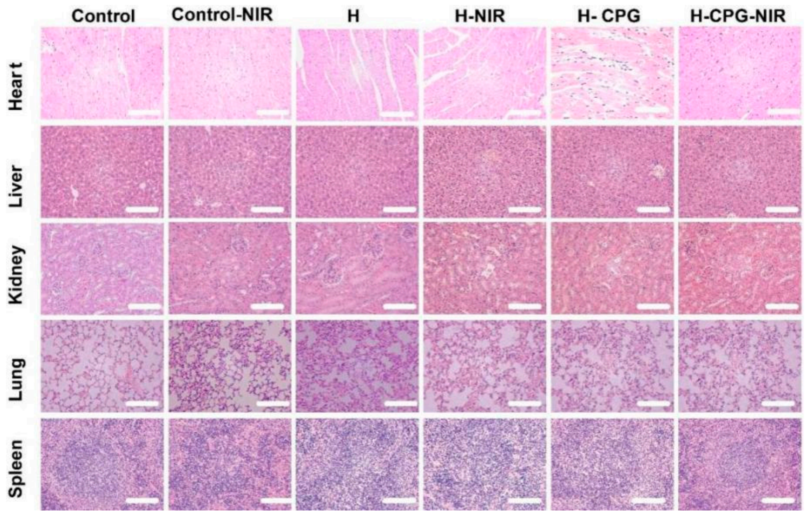

Figure S9. Histologic assessment of major organs in mice treated with various drugs by H&E staining. Scale bar: 200 μm.

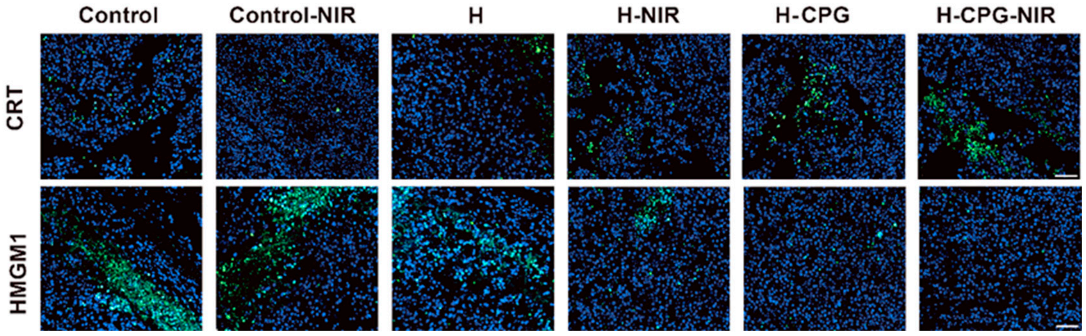

Figure S10. Expression of CRT and HMGB1 in tumor tissues after treatment with different materials. Scale bar: 100 μm.
